# An internal pilot study for a randomized trial aimed at evaluating the effectiveness of iron interventions in children with non-anemic iron deficiency: the OptEC trial

**DOI:** 10.1186/s13063-015-0829-4

**Published:** 2015-07-14

**Authors:** Kawsari Abdullah, Kevin E. Thorpe, Eva Mamak, Jonathon L. Maguire, Catherine S. Birken, Darcy Fehlings, Anthony J. Hanley, Colin Macarthur, Stanley H. Zlotkin, Patricia C. Parkin

**Affiliations:** Division of Pediatric Medicine, Department of Pediatrics, Pediatrics Outcomes Research Team, The Hospital for Sick Children, Room 109708, 10th Floor, 555 University Avenue, M5G 1X8 Toronto, ON Canada; Institute of Health Policy, Management and Evaluation, University of Toronto, 155 College Street Suite 425, M5T 3M6 Toronto, Canada; The Applied Health Research Centre, Li Ka Shing Knowledge Institute of St. Michael’s Hospital, 30 Bond Street, M5B 1W8 Toronto, ON Canada; Dalla Lana School of Public Health, University of Toronto, 155 College Street 6th floor, M5T 3M7 Toronto, Canada; Department of Psychology, The Hospital for Sick Children, 555 University Avenue, M5G 1X8 Toronto, ON Canada; Department of Pediatrics, St. Michael’s Hospital, University of Toronto, 30 Bond Street, M5B 1W8 Toronto, Canada; Child Health Evaluative Sciences, Hospital for Sick Children Research Institute, Peter Gilgan Centre for Research and Learning (PGCRL), 686 Bay Street, floor 10, room#109832, M5G 0A4 Toronto, ON Canada; Division of Developmental Pediatrics, Holland Bloorview Kids Rehabilitation Hospital, and Bloorview Research Institute, 150 Kilgour Road, M4G 1R8 Toronto, ON Canada; Department of Pediatrics, Faculty of Medicine, University of Toronto, 1 King’s College Circle, Medical Sciences Building, Room 2109, M5S 1A8 Toronto, Canada; Department of Nutritional Sciences, University of Toronto, FitzGerald Building, 150 College Street, Room 316, M5S 3E2 Toronto, ON Canada; Division of Endocrinology, Department of Medicine, Faculty of Medicine, University of Toronto, 1 King’s College Circle, Medical Sciences Building, Room 2109, M5S 1A8 Toronto, Canada; Research Institute, The Hospital for Sick Children, Peter Gilgan Centre for Research and Learning (PGCRL), 686 Bay Street, M5G 0A4 Toronto, ON Canada; Division of Gastroenterology, Hepatology and Nutrition, Department of Pediatrics, The Hospital for Sick Children and University of Toronto, 555 University Avenue, M5G 1X8 Toronto, Canada; Centre for Global Child Health, The Hospital for Sick Children, 555 University Avenue, M5G 1X8 Toronto, Canada

**Keywords:** Internal pilot study, Standard deviation, Sample size, Adherence

## Abstract

**Background:**

The OptEC trial aims to evaluate the effectiveness of oral iron in young children with non-anemic iron deficiency (NAID). The initial sample size calculated for the OptEC trial ranged from 112–198 subjects. Given the uncertainty regarding the parameters used to calculate the sample, an internal pilot study was conducted. The objectives of this internal pilot study were to obtain reliable estimate of parameters (standard deviation and design factor) to recalculate the sample size and to assess the adherence rate and reasons for non-adherence in children enrolled in the pilot study.

**Methods:**

The first 30 subjects enrolled into the OptEC trial constituted the internal pilot study. The primary outcome of the OptEC trial is the Early Learning Composite (ELC). For estimation of the SD of the ELC, descriptive statistics of the 4 month follow-up ELC scores were assessed within each intervention group. The observed SD within each group was then pooled to obtain an estimated SD (S_2_) of the ELC. Correlation (ρ) between the ELC measured at baseline and follow-up was assessed. Recalculation of the sample size was performed using analysis of covariance (ANCOVA) method which uses the design factor (1- ρ^2^). Adherence rate was calculated using a parent reported rate of missed doses of the study intervention.

**Conclusion:**

The new estimate of the SD of the ELC was found to be 17.40 (S_2_). The design factor was (1- ρ2) = 0.21. Using a significance level of 5 %, power of 80 %, S_2 =_ 17.40 and effect estimate (Δ) ranging from 6–8 points, the new sample size based on ANCOVA method ranged from 32–56 subjects (16–28 per group). Adherence ranged between 14 % and 100 % with 44 % of the children having an adherence rate ≥86 %. Information generated from our internal pilot study was used to update the design of the full and definitive trial, including recalculation of sample size, determination of the adequacy of adherence, and application of strategies to improve adherence.

**Trial registration:**

ClinicalTrials.gov Identifier: NCT01481766 (date of registration: November 22, 2011).

**Electronic supplementary material:**

The online version of this article (doi:10.1186/s13063-015-0829-4) contains supplementary material, which is available to authorized users.

## Update

### Background

This update reports the findings from an internal pilot study that aimed to obtain parameter estimates for recalculation of the sample size of the OptEC trial (Optimizing Early Child Development in the Primary Care Practice Setting) and also assess the adherence rate of the participants in the internal pilot study.

The OptEC trial aims to evaluate the effectiveness of oral iron plus nutritional guidance over placebo plus nutritional guidance in children with non-anemic iron deficiency (NAID) in improving their developmental, hematological and behavioral outcomes. Sample size for the OptEC trial ranged from 112–198 (N_a_) participants (using a standard deviation, S_1_ = 15 and a range of effect estimates, Δ of 6–8 points). The sample size was calculated using the t-test. A detailed description of the OptEC trial has previously been published [1].

The design of the OptEC trial includes an internal pilot study [2]. The rationale for conducting an internal pilot for the OptEC trial was three fold. First, there was uncertainty regarding the parameters used to calculate the sample size for the OptEC trial [3]. The estimates used were obtained from previous trials which had different study conditions, for example, different population, small numbers of centers and different treatment duration. Thus, prior estimates may not be representative of the current trial. Inaccurate estimates may lead to an unnecessarily large trial or a trial not large enough to have sufficient power for detection of a clinically relevant treatment effect. Data generated from internal pilots are used to obtain more reliable estimates of parameters for recalculation of sample size of clinical trials [3–6].

Second, we aimed to recalculate the sample size for the OptEC trial using the method known as the ANCOVA (analysis of covariance). One advantage of the ANCOVA method is that it accounts for the correlation between the baseline and follow-up assessment of the primary outcome, in the calculation of the sample size [7]. Thus the sample size calculated using this method will have the same power as the t-test but will require fewer subjects [7]. In order to use the ANCOVA method, we need to know the correlation between the baseline and follow-up assessment of the primary outcome of the OptEC trial which we intend to calculate using our internal pilot data.

Third, evidence suggests low level of adherence to interventions in clinical trials investigating the efficacy of oral iron interventions [8]. A review of adherence in primary school children showed the adherence rate to oral iron to range between 50 %-90 % [9]. Lack of adherence may decrease the probability of detecting treatment differences and affect the interpretation of observed differences. However, partial adherence is usually sufficient to evaluate the effectiveness of an intervention. According to previous research, if 40 % of the participants in a randomized trial have at least 90 % of adherence then the effectiveness assessment process remains unimpaired [10]. Data from an internal pilot study can be used to check the level of adherence in participants of clinical trials [11–13]. If adherence is found to be less then desired, then strategies can be implemented to improve adherence.

The overall aim of the internal pilot was to inform the design of the full and definitive trial. Thus, the objectives of the internal pilot study were (1) to obtain a reliable estimate of the standard deviation (S_2_) of the primary outcome of the OptEC trial; (2) to obtain the correlation between the baseline and follow-up measurement of the primary outcome; (3) to recalculate the sample size (N_r_) of the OptEC trial using the estimates generated from the internal pilot; and (4) to assess the adherence rate and causes of non-adherence in children enrolled in the pilot study.

At the end of the pilot study, if necessary the sample size will be recalculated and compliance measures may need to be enhanced. Otherwise, the OptEC trial will continue following the protocol as previously reported [1]. During the collection and analysis of data for the internal pilot study the recruitment of the trial continued.

## Methods

The internal pilot study is an integral part of the OptEC trial, which consisted of the first few participants enrolled in the trial. Hence, it follows the same design and conduct of the main trial. In the following sections we describe methods that are particularly relevant to the internal pilot study as recommended by Thabane et al. for reporting of pilot study results [14].

### Design

The OptEC trial, hence the internal pilot study was a multi-site, pragmatic, placebo controlled, superiority randomized trial [1].

### Participants

Eligibility criteria for participation in the internal pilot were the same as those for the OptEC trial. Inclusion criteria: children with NAID [hemoglobin >110 g/L, serum ferritin < 14 μg/L and C-reactive protein (CRP) <10 mg/L]; and age 12 to 40 months. Exclusion criteria: CRP level >10 mg/L, previously diagnosed developmental disorder, genetic, chromosomal or syndromic condition, chronic medical conditions (except asthma and allergies), including chronic anemia, recent oral iron supplementation or treatment, gestational age less than 35 weeks, low birth weight less than 2500 grams, attending the office for an acute illness, any contraindications to receiving elemental iron, the use of any natural health product containing the same medicinal ingredient(s) as the investigational product, English not spoken to the child in the home or in a child care setting.

### Intervention and control

Children are randomized to receive either oral iron treatment (6 mg elemental iron/kg/day) or placebo (equivalent volume) twice daily for four months. Children in both the oral iron and placebo groups are also given nutritional guidance to improve iron intake which includes recommendations on the varied sources of high iron containing foods, foods that increase and inhibit iron absorption, and dietary habits that may prevent iron deficiency (such as - maximum daily cow’s milk intake, limiting the intake of juice). Concomitant interventions permitted include over the counter multivitamins which do not contain iron; those prohibited include additional over the counter iron and prescription iron.

### Primary outcome and measures

The primary outcome for the OptEC trial is the Early Learning Composite (ELC) assessed using the Mullen Scales of Early Learning (MSEL) [15]. The MSEL measures five distinct developmental skills, gross motor and four “cognitive” skills - fine motor, visual reception, receptive language, and expressive language. The four cognitive skills are summarized and converted into age adjusted normalized ELC, which has a mean of 100 and a standard deviation of 15. Developmental assessment using the MSEL is performed at baseline and after 4 months of intervention by a trained psychometrist under the supervision of a registered psychologist. All individuals involved with data collection, entry and analysis are blind to the group assignment.

### Collection of other variables

Baseline data collection for the internal pilot included age and sex of child, birth weight, weight, length/height, maternal ethnicity and education, family income and some nutritional behavior characteristics (total duration of breastfeeding, volume of cow’s milk intake and current bottle feeding). These data were collected using a parent-completed, standardized data collection form based on questions used in the Canadian Community Health Survey. We dichotomized family income based on median income of families in the city of Toronto [16].

Adherence related data were collected during the 4 month follow-up visit which included reasons for non-adherence, adverse effects and a parent reported weekly rate of days the study intervention was not taken.

### Sample size for the internal pilot study

The minimum size for an internal pilot study should be at least 10 subjects per treatment group, for a two group randomized trial [4]. The pre-planned sample size for the OptEC trial ranged from 112–198 (approximately 150 subjects) [1]. The first 15 participants per treatment group enrolled in the OptEC trial were considered as the internal pilot study (total *n* = 30).

### Statistical methods for the internal pilot study

For estimation of parameters for sample size recalculation, descriptive statistics of the 4 month follow-up developmental data were assessed within each treatment group. The observed standard deviation of the ELC score within each treatment group was pooled to obtain an estimated standard deviation (S_2_) of the ELC score (Additional file 1 shows the formula for calculating the pooled standard deviation) [3, 17].

Recalculation of the sample size was performed using the analysis of covariance (ANCOVA) method which uses the design factor (or variance deflation factor) to calculate the sample size [7]. The design factor is (1- ρ^2^), where ρ is the correlation between the baseline and follow-up measurement of the primary outcome. The ANCOVA uses a two-step method to calculate sample size [7]. Step 1: First, a sample size is determined using the two sample independent t-test and the new estimate of the SD (S_2_). Step 2: Then, the correlation (ρ) between the baseline and 4 month follow-up measure of the ELC is calculated using Pearson’s correlation. This value is used to determine the design factor (1- ρ^2^). The value of the sample sizes calculated using the t-test is then multiplied by the design factor (1-ρ^2^) to produce the number of participants (N_r_) required by the ANCOVA method.

The reasons for non-adherence were identified and summarized. Adherence rate was calculated using a method proposed by Klerk et al. [18]. In this method, summaries such as - the total number of days per week the child received the study intervention, the length of the monitored interval and the over-all percentage of the study intervention taken during the monitored interval was used to calculate a rate of adherence. Furthermore, adherence rate was described by placing participants into broad bands of adherence. The adherence bands corresponded to the number of days per week the participants received the intervention, such as, ≥6 days correspond to 86 % - 100 % adherence; 4–5 days to 57 % - 85 %; 2–3 days to 28 % - 56 %; and ≤1 day to 0 – 27 %. The percentage of children in each band was also determined.

For the purpose of the internal pilot study, the two treatment groups were identified as group A and group B by a third party, so as to keep group assignment blinded to all persons associated with the internal pilot study. Statistical analyses were performed using SAS software version 9.1 (SAS Institute, Cary NC) and sample size calculation was performed using the Vanderbilt University, Department of Biostatistics, power and sample size calculator [19].

### Ethics approval

The OptEC trial was granted ethics approval by The Hospital for Sick Children Research Ethics Board (REB File No.: 1000027782) on May 10, 2012 and approval is renewed annually by the REB. Written informed consent is obtained from parents of all child participants (including the internal pilot study) prior to any data collection.

#### Criteria for success of the internal pilot study

Pre-specified criteria for success [14] for recalculation of the sample size were as follows: if the estimated SD S_2_ ≤ 15, the trial would continue as planned, that is the initial sample size (Na = 112–198) will not change. However, if the estimated SD, S_2_ > 15, then the sample size will be recalculated [3]. For adherence related data, we projected that 40 % of the participants in the internal pilot study will have 86 % - 100 % of adherence (the highest band of adherence). There was no stopping rule for the internal pilot study that would halt the OptEC trial.

## Results

### Participant flow and baseline characteristics

**Fig. 1:**
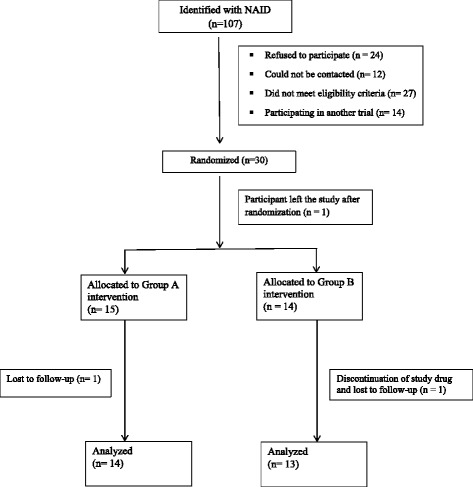
Participant flow diagram for the internal pilot study

A total of 107 children with NAID were identified between June, 2012 and June, 2014. Of these children ultimately a total of 30 were randomized to the two intervention groups (see Fig. 1: participant flow diagram). Table 1 depicts the baseline characteristics of the participants in the internal pilot of the OptEC trial.

**Table 1:** Baseline characteristics of the children in the internal pilot study (*N* = 30)

Variables names	Descriptive data	
Age of child (months)		23.5 (6.95)^a^
Sex of child	Male	15 (50)^b^
	Female	15 (50)^b^
zBMI of child		0.49 (0.93)^a^
Birth weight (kg)		3.3 (0.43)^a^
Maternal ethnicity	European	20 (74.07)^b^
	Non-European	7 (25.93)^b^
Maternal education	University/Diploma	19 (70.37)^b^
	College/non-university Diploma	5 (18.52)^b^
	High School	2 (7.41)^b^
Family income^c^	Above median income	22 (81.48)^b^
	Below median income	5 (18.52)^b^
Duration of breastfeeding (months)		14.56 (4.97)^a^
Volume of cow’s milk (cups/day)		2.46 (1. 20)^b^
Currently use bottle	Yes	11 (42.31)^b^
	No	15 (57.69)^b^
Serum ferritin (μg/L)		8.90 (2.47)^a^
Hemoglobin (g/L)		120.48 (9.34)^a^

^a^mean (SD)

^b^n (%)

^c^Income based on median income of families in the city of Toronto [11]

### Estimation of the standard deviation (S_2_) and recalculation of the sample size

Table 2 shows the SD of the follow-up developmental data. The SD of the ELC in group A and group B was 21.17 and 12.05, respectively. These two values were pooled to calculate an estimated SD, S_2_ = 17.4. However, we observed a large difference between the SDs of the two treatment groups. An F-test was performed, where we were unable to reject the null hypothesis that the variances were equal (F value = 3.09 and *p* = 0.06) [17]. Therefore, the pooled estimate of the SD (S_2_) was used to recalculate the sample size.

**Table 2:** Follow-up developmental data of the participants in the internal pilot study

Developmental score	Group A *n* = 14	Group B *n* = 13
	SD	SD
Early Learning Composite (ELC)	21.17	12.05

*SD* standard deviation

Using a significance level of 5 %, a power of 80 %, SD value of S_2_ = 17.4 and clinically meaningful effect estimate ranging from 6–8 points, a range of sample sizes were calculated first using the t-test method (see Table 3). Correlation (ρ) between the ELC measured at baseline and 4 month follow-up was 0.89. Hence, the design factor for assessment of the ELC was, (1- ρ^2^) = 0.21. Then, following the ANCOVA method for calculating sample size, the design factor was multiplied to the values of the sample size calculated using the t-test and a range of sample sizes for the OptEC trial was recalculated (N_r_ = 32–56) (see Table 3).

**Table 3:** Range of sample sizes recalculated using internal pilot data

Calculated sample size	α = 5 %; 1-β = 80 %; and S_2_ = 17.4		
	Δ = 8	Δ = 7	Δ = 6
Using t-test	150	196	266
	Multiplication by the design factor (1- ρ^2^) = 0.21		
Using ANCOVA	32	42	56

*α* significance level, *1-β* power, *S*_*2*_ SD, *Δ* effect estimate

### Assessment of adherence rate and causes of non-adherence

The main reasons for non-adherence among the internal pilot sample were - the study drug takes too long to administer, is too messy, child did not like it, too difficult to administer and forgot to give the study drug. Adverse effects reported by the parents included vomiting (19 %), staining of teeth (34 %), constipation (38 %), loose stool (35 %) and passage of black stool (46 %). We found adherence to the study intervention to range between 14 % and 100 %. We then identified the number and proportion of children in each adherence band (see Table 4). The highest adherence band (86 % - 100 %) had a total of 12 (44 %) children.

**Table 4:** Adherence rates of participants in the internal pilot study (*N* = 27)

Bands of adherence rate (%)	Number of participants in each band (n)	Percentage of participants in each band (%)
86 - 100	12	44.44
57 - 85	6	22.22
28 - 56	2	7.41
0 - 27	7	25.93

## Discussion

This internal pilot study allowed a data-driven recalculation of the sample size for a randomized controlled trial. If knowledge about the standard deviation of the primary endpoint is weak, this type of approach is superior to an ordinary fixed sample design, because the initial sample size can be appropriately adjusted.

According to our first objective, an estimate of the SD (S_2_ = 17.4) of the primary endpoint was determined by pooling the observed SD in the two treatment groups (Table 2). This estimate was found to be larger than the SD (S_1_ = 15) we used to calculate the initial sample of the OptEC trial. Hence, as stated in our criteria of success, the new estimate of the SD was used to recalculate the sample size.

Our second objective, recalculation of the sample size involved the application of the ANCOVA method [7]. This method requires the estimation of the design factor that accounts for the correlation between the baseline and follow-up assessment of the primary outcome, our third objective. We determined the design factor [(1- ρ2) = 0.21] using data from the internal pilot study. The sample sizes calculated using the ANCOVA method ranged between 32–56 subjects which will have the same power as the sample sizes calculated using the t-test (150–266), despite being considerably smaller (see Table 3) [7]. In situations where recruitment of participants is a challenge, application of the ANCOVA method is a valid approach to minimize the sample size in clinical trials without affecting the power of the trial. In our OptEC trial, we aim to enroll a total of 56 participants (28/group), in order to detect a treatment difference of 6 points in the ELC score.

Our fourth objective was to determine the adherence rate in our internal pilot study. According to previous evidence ≥90 % of adherence in 40 % of participants was shown to be ideal for effectiveness assessment [10]. Since our adherence assessment was based on the number of days per week the child received the study intervention, our study team modified this criteria for success to ≥86 % adherence, as this corresponds to ≥6 days in a week. In our study we found 44 % of participants having an adherence rate ≥86 % (see Table 4). We believe this level of adherence will be sufficient to meet the objective of the OptEC trial to assess the effectiveness of the study intervention.

Because some children had adherence rate as low as 14 %, we have implemented strategies to improve adherence, such as - informing parents on possible adverse effects, decreasing the dose of the study intervention when children experience adverse effects like vomiting and diarrhea, counseling parents on minimizing the difficulty of giving the study intervention and motivating parents on continuing the study intervention when children experience non-harmful and reversible adverse effects like passage of black stool and staining of teeth. This information has been incorporated into a participant information handout which is given to parents when they agree to enroll their child in the OptEC trial.

At the end of the trial, participants in the internal pilot study will contribute to the overall sample and outcome analysis of the full trial. According to internal pilot methodology, this inclusion will have minimal impact on the significance level of the test of treatment effect [4]. The adherence rate reported in this study may be an overestimate of the actual adherence, due to the fact that it was based on parent reported measures.

## Conclusion

Information generated from our internal pilot study was used to update the currently ongoing OptEC trial. It not only provided us with a more reliable and representative estimate of the SD of the primary outcome, but also provided us with the necessary parameters needed to calculate our sample size using the ANCOVA method. Furthermore, our study depicts other valid uses of internal pilot data such as the estimation of adherence rate. The internal pilot design is one of the methods suggested by the StaR (Standards for Research in Child Health) standard development groups to overcome challenges faced when attempting to derive sample size estimates in pediatric research [20]. The data generated from our internal pilot can inform future trials to be performed in similar population, particularly those in developed country settings.

## Additional file

Additional file 1:
**Formula to calculate pooled standard deviation.**

## Abbreviations

OptEC: Optimizing Early Child Development in the Primary Care Practice Setting
NAID: Non-anemic iron deficiency
SD: Standard deviation
ANCOVA: Analysis of covariance
MSEL: Mullen scale of early learning
ELC: Early learning composite
CRP: C-reactive protein
