# Supplementary material for: An internal pilot study for a randomized trial aimed at evaluating the effectiveness of iron interventions in children with non-anemic iron deficiency: the OptEC trial
Source: Trials. 2015 Jul 14;16:303. doi: 10.1186/s13063-015-0829-4 (PMC4501099; doi:10.1186/s13063-015-0829-4)
Supplement: Additional file 1: — Formula to calculate pooled standard deviation. [file 13063_2015_829_MOESM1_ESM.pdf]

Appendix 1:

Formula to calculate pooled standard deviation (17):

$(S_2)^2 = [(n_1 – 1)\ S_A^2 + (n_2 – 1)\ S_B^2] / (n_1 + n_2 – 2)$

S_2 = pooled SD

$S_{A\ =}$ observed SD in group A

$S_{B\ =}$ observed SD in group B

$n_{1\ =}$ sample size for group A

n_2 = sample size for group B
